# Supplementary material for: Ciprofloxacin and Levofloxacin as Potential Drugs in Genitourinary Cancer Treatment—The Effect of Dose–Response on 2D and 3D Cell Cultures
Source: Int J Mol Sci. 2021 Nov 4;22(21):11970. doi: 10.3390/ijms222111970 (PMC8584631; doi:10.3390/ijms222111970)
Supplement: Supplementary file 1 [file ijms-22-11970-s001.zip › Figure Legends.pdf]

Figure S1. Cytotoxic properties of ciprofloxacin and levofloxacin on bladder cell lines. Results obtained using MTT assay after 24 and 48 hours incubation with both drugs. Decreased viability of all tested cell lines were observed after treatment with increasing ciprofloxacin and levofloxacin concentrations. Almost in all cases 48 hour incubation with drugs resulted in lower cell viability. Comparison of both drugs on non-malignant bladder cell line showed advantage of ciprofloxacin. SV-HUC-1 – non-malignant human urothelium; T24 - human bladder cancer; * - $p<0.05$; ** - $p<0.01$, *** - $p <0.001$, **** -$p<0.0001$, ns – not significant.

Figure S2. Cytotoxic properties of ciprofloxacin and levofloxacin on prostate cell lines. Results obtained using MTT assay after 24 and 48 hours incubation with both drugs. Decreased viability of all tested cell lines were observed after treatment with increasing ciprofloxacin and levofloxacin concentrations. Almost in all cases 48 hour incubation with drugs resulted in lower cell viability. Comparison of both drugs on non-malignant prostate cell line showed advantage of ciprofloxacin after 48 hour incubation with drugs. DU-145 - human prostate cancer; RWPE-1 – non-malignant human prostate epithelium; * - $p<0.05$; ** - $p<0.01$, *** - $p <0.001$, **** - $p<0.0001$.

Figure S3. Caspases activity analysis. Analysis of caspase 3/7 and 9 activity on all forur tested cell lines treated with LC50 concertation of ciprofloxacin and levofloxacin obtained after 24h incubation. SV-HUC-1 - normal human urothelium; T24 - human bladder cancer; DU-145 - human prostate cancer; RWPE-1 - normal human prostate epithelium; * - $p<0.05$; ** - $p<0.01$, *** - $p <0.001$, **** -$p<0.0001$.
