# Supplementary material for: Ciprofloxacin and Levofloxacin as Potential Drugs in Genitourinary Cancer Treatment—The Effect of Dose–Response on 2D and 3D Cell Cultures
Source: Int J Mol Sci. 2021 Nov 4;22(21):11970. doi: 10.3390/ijms222111970 (PMC8584631; doi:10.3390/ijms222111970)
Supplement: Supplementary file 1 [file ijms-22-11970-s001.zip › Table S1.pdf]

Supplementary Table S1. BD FACS Canto II detectors configuration

Laser	Detector	Filter	Mirror
**488 nm - 20mW (Blue)**	**A**	780/60	735LP
	**B**	670LP	655LP
	**C**	-	610LP
	**D**	585/42	556LP
	**E**	530/30	520LP
	**F**	488/10	**-**
**633 nm – 17mW (Red)**	**A**	780/60	735LP
	**B**	-	685LP
	**C**	660/20	-
**405nm – 30mW (Violet)**	**A**	510/50	502LP
	**B**	450/50	-
