# Supplementary material for: Ciprofloxacin and Levofloxacin as Potential Drugs in Genitourinary Cancer Treatment—The Effect of Dose–Response on 2D and 3D Cell Cultures
Source: Int J Mol Sci. 2021 Nov 4;22(21):11970. doi: 10.3390/ijms222111970 (PMC8584631; doi:10.3390/ijms222111970)
Supplement: Supplementary file 1 [file ijms-22-11970-s001.zip › Table S2.pdf]

Supplementary Table S2. Lethal concentrations (LC, µg/ml values) calculated for ciprofloxacin and levofloxacin after 24 and 48 hours incubation with SV-HUC-1 (normal human urothelium), T24 (human bladder cancer), DU-145 (human prostate cancer) and RWPE-1 (normal human prostate epithelium) cell lines. With asterixis we marked values that were not achieved in MTT assay and were calculated theatrically form curve equation.

**Ciprofloxacin**					
	LC [µg/ml]	SV-HUC1	T24	RWPE-1	DU-145
24h	LC10	55.59	5.11	21.24	31.56
	LC50	264.54	86.84	155.56	165.28
	LC90	1258.99*	1510.84*	1519.80*	865.45*
48h	LC10	37.58	20.20	25.02	15.60
	LC50	171.87	39.94	46.23	81.18
	LC90	786.07	264.70	215.00	427.65
**Levofloxacin**					
24h	LC10	202.66	37.59	81.72	31.24
	LC50	869.30*	414.34	493.68	334.25
	LC90	1536.00*	4670.00*	2982.30*	2204.00*
48h	LC10	62.00	28.52	37.45	15.33
	LC50	506.44	143.71	177.87	151.94
	LC90	950.89*	730.63	845.04*	1505.81*
