# Supplementary material for: Ciprofloxacin and Levofloxacin as Potential Drugs in Genitourinary Cancer Treatment—The Effect of Dose–Response on 2D and 3D Cell Cultures
Source: Int J Mol Sci. 2021 Nov 4;22(21):11970. doi: 10.3390/ijms222111970 (PMC8584631; doi:10.3390/ijms222111970)
Supplement: Supplementary file 1 [file ijms-22-11970-s001.zip › Table S3.pdf]

Supplementary Table S3. Results of cell cycle analysis after incubation with ciprofloxacin and levofloxacin (in percentage).

			**G0/G1**	**S**	**G2/M**
**Ciprofloxacin**	SV-HUC-1	**Control**	39.2 ± 0.9	37,3 ± 5.0	25,2 ± 5.0
		**LC_{10}**	37.6 ± 3.3	36.5 + 3.4	25.9 ± 5.8
		**LC_{50}**	31.2 ± 10.3	44.9 ± 7.9	24.1 ± 3.2
		**LC_{90}**	28.7 ± 3.9	48.6 ± 7.5	21.3 ± 1.9
	T24	**Control**	72.4 ± 16.5	19.8 ± 9.2	9.9 ± 2.4
		**LC_{10}**	69.0 ± 17.9	21.7 ± 9.8	11.5 ± 2.7
		**LC_{50}**	56.6 ± 11.5	31.4 ± 8.2	14.0 ± 0.8
		**LC_{90}**	41.7 ± 1.5	40.1 ± 2.6	18.8 ± 1.5
	RWPE-1	**Control**	62.2 ± 9.2	25.5 ± 8.4	10.0 ± 1.7
		**LC_{10}**	70.2 ± 6.6	19.9 ± 6.8	7.5 ± 0.6
		**LC_{50}**	56.0 ± 3.4	28.8 ± 4.3	14.2 ± 2.3
		**LC_{90}**	65.0 ± 6.3	25.3 ± 2.9	7.5 ± 2.5
	DU-145	**Control**	44.0 ± 8.6	38.2 ± 7.5	17.0 ± 1.3
		**LC_{10}**	37.6 ± 7.1	42.2 ± 6.5	18.6 ± 1.4
		**LC_{50}**	46.6 ± 2.6	31.5 ± 1.0	19.1 ± 1.6
		**LC_{90}**	43.2 ± 1.7	34.8 ± 4.8	18.2 ± 1.4
**Levofloxacin**	SV-HUC-1	**Control**	36,8 ± 0.9	38.5 ± 1.7	27.2 ± 5.1
		**LC_{10}**	39.4 ± 5.8	37.1 ± 5.8	27.2 ± 4.9
		**LC_{50}**	30.2 ± 3.7	46.0 ± 8.7	25.5 ± 3.8

		**LC_{90}**	34.0 ± 4.8	46.0 ± 7.6	22.7 ± 2.3
	T24	**Control**	56.7 ± 15.1	26.6 ± 10.5	16.1 ± 4.4
		**LC_{10}**	54.7 ± 12.9	29.5 ± 8.5	16.3 ± 3.2
		**LC_{50}**	50.0 ± 0.9	33.0 ± 0.8	16.0 ± 2.6
		**LC_{90}**	42.6 ± 2.7	36.3 ± 5.8	18.9 ± 5.4
	RWPE-1	**Control**	59.5 ± 5.6	28.1 ± 4.9	10.5 ± 1.9
		**LC_{10}**	69.6 ± 7.2	21.4 ± 4.9	7.9 ± 2.7
		**LC_{50}**	60.9 ± 9.6	22.9 ± 5.9	15.6 ± 2.9
		**LC_{90}**	56.6 ± 2.4	27.7 ± 1.6	14.6 ± 1.2
	DU-145	**Control**	39.8 ± 12.7	44.0 ± 13.7	14.2 ± 1.4
		**LC_{10}**	41.9 ± 13.7	42.4 ± 15.0	15.5 ± 3.6
		**LC_{50}**	53.9 ± 5.8	27.0 ± 1.8	16.9 ± 4.7
		**LC_{90}**	24.7 ± 0.9	56.7 ± 0.9	14.5 ± 1.2
