# Supplementary figures and images for: Ciprofloxacin and Levofloxacin as Potential Drugs in Genitourinary Cancer Treatment—The Effect of Dose–Response on 2D and 3D Cell Cultures
Source: Int J Mol Sci. 2021 Nov 4;22(21):11970. doi: 10.3390/ijms222111970 (PMC8584631; doi:10.3390/ijms222111970)

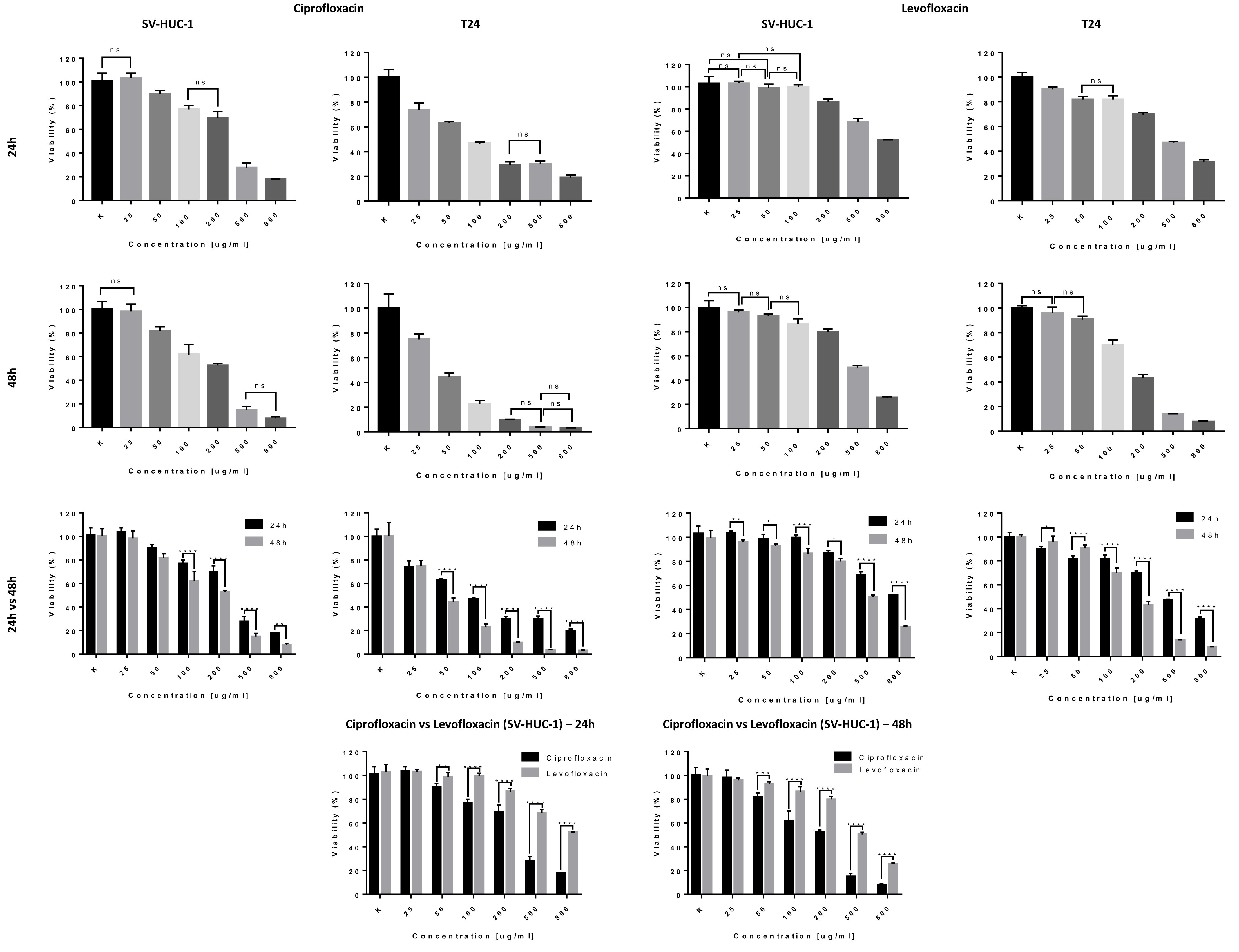

Supplement: Supplementary file 1 [file ijms-22-11970-s001.zip › Supp.Fig.1.tif]

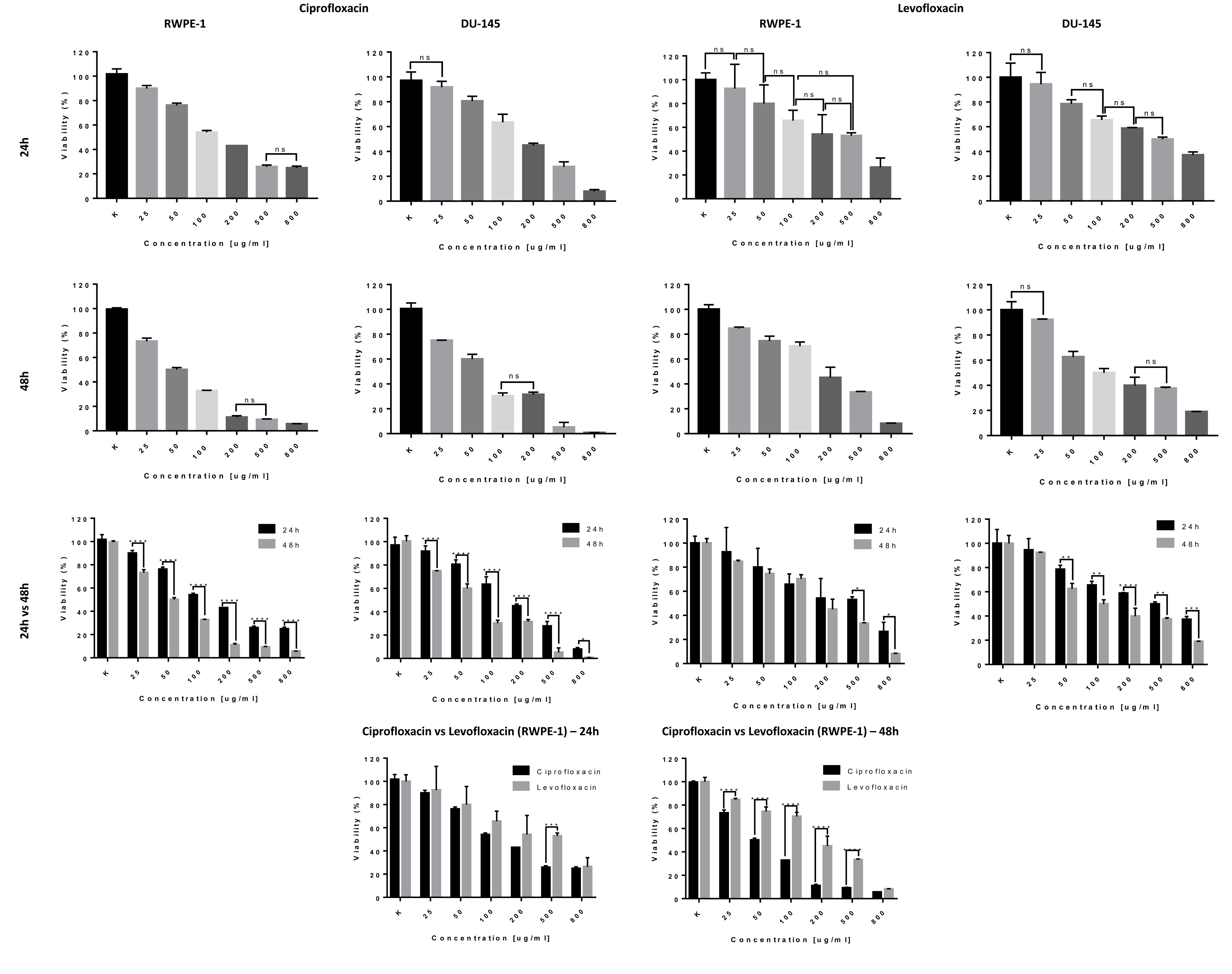

Supplement: Supplementary file 1 [file ijms-22-11970-s001.zip › Supp.Fig.2.tif]

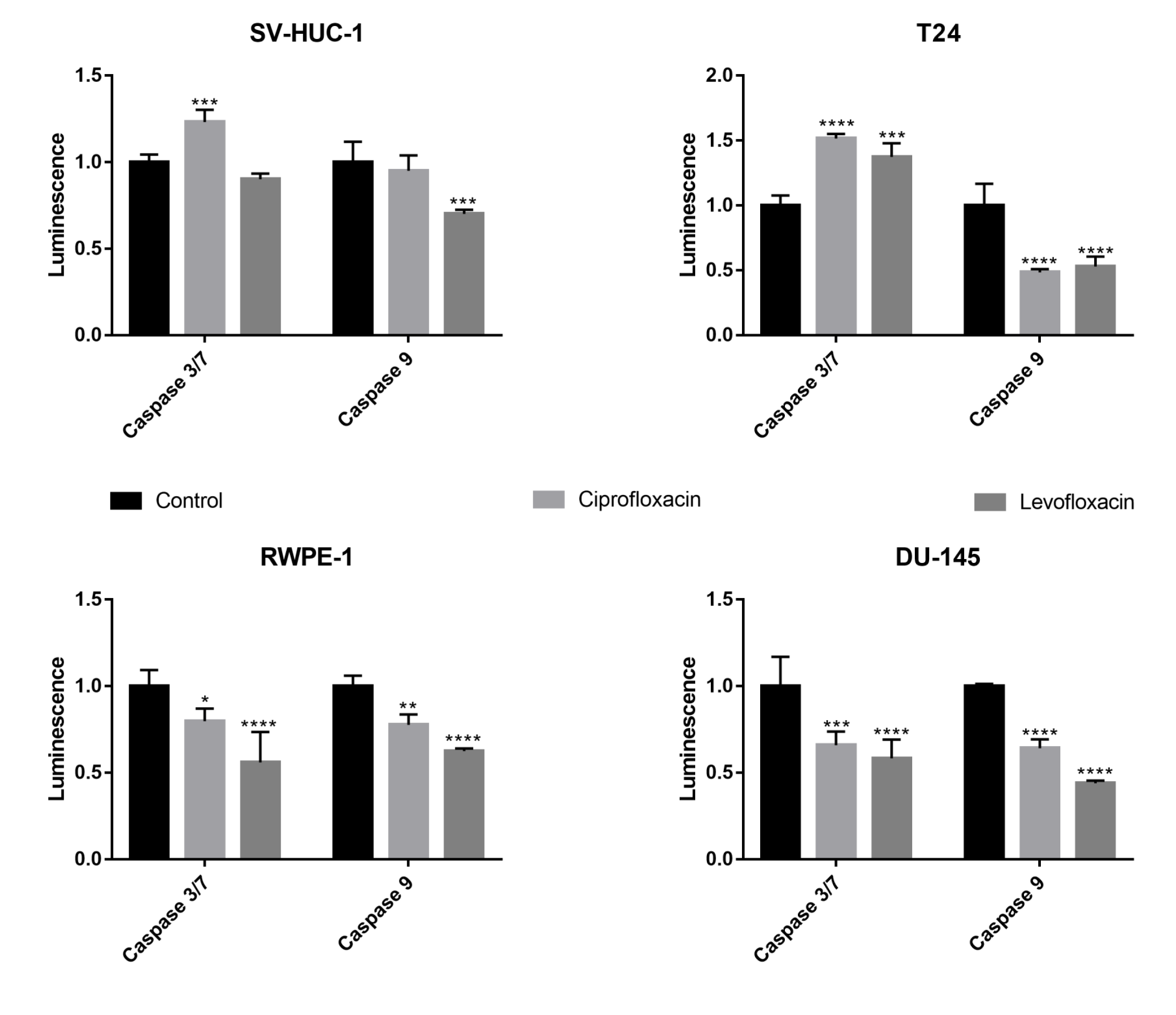

Supplement: Supplementary file 1 [file ijms-22-11970-s001.zip › Supp.Fig.3.tif]
